# Recruitment strategies for Turkish immigrants in dementia care research: a scoping review

**DOI:** 10.1186/s12877-025-06031-3

**Published:** 2025-06-05

**Authors:** Esma Inam, Seda Güney, Martina Roes

**Affiliations:** 1 German Center for Neurodegenerative Diseases (DZNE), Witten Site, Germany; 2 Faculty of Sociology, University of Duisburg/Essen, Duisburg, Germany; 3 School of Nursing, Koç University, Istanbul, Turkey; 4 Faculty of Health, Department of Nursing Science, University of Witten/Herdecke, Witten, Germany

**Keywords:** Caregiver, Dementia, Recruitment, Turkish

## Abstract

**Background:**

Among the ethnic minority groups in Germany, Turkish immigrants represent the largest community. At the same time, this target group is underrepresented in dementia care research, and there are unique challenges regarding the recruitment of this group for studies. Increasing the involvement of Turkish immigrants in dementia care research is essential for reducing dementia-related inequalities. The aim of this scoping review is to describe strategies for recruiting Turkish immigrants living with dementia and their caregiving relatives for dementia care research and to identify related recruitment challenges.

**Methods:**

We conducted a scoping review including articles from peer-reviewed journals applying the methodological framework of Arksey and O'Malley and the PCC framework (P = Population, C = Concept, C = Context). We searched the MEDLINE, PsycINFO, CINAHL, and Scopus databases as well as the literature written in the German language in local university databases. There were no restrictions on publication dates or study types. Additionally, the references of the identified articles were manually searched, and relevant articles were added. The content analysis was used to synthesize the findings.

**Results:**

In total, 651 articles were screened, 15 of which were included in the review. Nine of the 15 articles were focused on the involvement of caregiving relatives, and six of the included articles were focused on people with dementia. The choice of recruitment strategies depended on the specific target group. Five main categories were identified based on the characteristics of the recruitment strategies implemented: Access to health environment, inclusive practices and cultural considerations, face-to-face interactions, technology-mediated access and engagement events. People with dementia were recruited predominantly through senior centres, hospitals, or home care providers; referrals from physicians; and databases/registries. The involvement of bilingual staff was a crucial strategy for achieving greater participation. Caregiving relatives were recruited mainly through dementia-related community organizations, settings, and social networks and through the involvement of bilingual staff. While facilitators play an essential role in engaging caregiving relatives, language and cultural barriers remain the most important barriers.

**Conclusions:**

The persistent presence of language and cultural barriers requires a culturally sensitive recruitment approach to increase the involvement of Turkish immigrants living with dementia and their caregiving relatives in dementia care research.

**Supplementary Information:**

The online version contains supplementary material available at 10.1186/s12877-025-06031-3.

## Background

Dementia is a global health issue that affects individuals and their families worldwide. As the prevalence of dementia continues to increase, there is a growing need for research aimed at understanding the unique but underresearched experiences and needs of diverse populations [1]. Among these underresearched populations, Turkish immigrants constitute a significant demographic group in several regions, particularly in Europe [2]. According to the Turkish Ministry of Foreign Affairs, more than 6.5 million Turkish citizens live abroad, and approximately 5.5 million have settled in the countries of Western Europe, of which more than 3 million live in Germany [3]. However, there is a lack of research on recruitment strategies for Turkish immigrants living with dementia and their caregiving relatives in dementia care studies.

Caring for individuals with dementia within the Turkish immigrant community presents distinct cultural, linguistic, and socioeconomic challenges that require targeted interventions and support [4]. Nevertheless, there are language- and culture-related difficulties in effectively recruiting and involving Turkish immigrants in dementia care studies [5]. The attitudes of Turkish immigrants towards language usage highlight a noticeable and somewhat anticipated contrast between linguistic choices in domestic and professional spheres. While many Turkish immigrants opt for the host country's language in the workplace, the Turkish language takes precedence in their homes. The selection of language for staying informed seems evenly distributed among participants, but there is a clear inclination towards using Turkish for entertainment purposes. Turkish immigrants consistently rate their proficiency in Turkish as high [6].

Emphasizing the need to account for cultural and linguistic factors while recruiting Turkish immigrants with dementia is crucial [7]. Understanding the experiences and needs of caregiving relatives can also inform recruitment strategies by addressing their specific concerns and providing appropriate support [8]. The diversity of their experiences impacts communication, care-giving methods, and perceptions of dementia, necessitating tailored strategies to effectively engage and support this specific population in research endeavours. By comprehensively examining the current state of recruitment methods, we sought to identify successful recruitment approaches and facilitators of and barriers to recruitment of Turkish immigrants with dementia and their caregiving relatives for research and to offer recommendations for future dementia-care research projects.

Some studies have highlighted language barriers, limited information about dementia and available services, cultural and family structures, and mistrust towards healthcare providers as significant hurdles in recruitment [5, 9]. Conversely, healthcare professionals in healthcare institutions with multicultural teams and professionals of the same ethnicity as the care beneficiaries have been identified as facilitators for recruiting caregiving relatives [10, 11]. The most successful approach for enhancing the recruitment and retention of individuals with dementia across diverse cultural groups has been identified as involving the establishment of strong community relationships and capacity building in communities [12].

However, to date, a detailed analysis of the recruitment of Turkish immigrants to become involved in dementia care research projects, either as participants or coresearchers, has not been performed. Therefore, this scoping review aims to fill this gap by examining recruitment strategies for this specific population conducted in dementia care studies. It addresses a significant void in the literature, as there is a shortage of comprehensive reviews specifically focusing on recruitment strategies for Turkish immigrants living with dementia and their caregiving relatives. By synthesizing the literature on recruitment strategies and considering the unique needs and challenges faced by this population, this review can offer valuable insights for researchers and practitioners working with Turkish immigrants with dementia and their caregiving relatives.

## Methods

We conducted a scoping review to identify strategies for recruiting Turkish people living with dementia (PLWD) and their caregiving relatives living in countries other than their home countries. We provide a comprehensive overview of the methods used in applied dementia care research studies. Scoping reviews are used to identify the key concepts of a research area, establish working definitions, or delineate the substantive boundaries of a topic [13].

The methodological framework of Arksey and O'Malley (2005) was utilized to conduct this scoping review. Arksey and O'Malley built a framework including six steps for a scoping review: (1) identification of the research question, (2) identification of the relevant studies, (3) study selection/screening process, (4) data extraction, (5) compilation, summary and reporting of the results, and (6) consulting (optional). The PRISMA Scoping Reviews Extension (PRISMA-ScR) checklist will also be used to enable transparent and comprehensive reporting of reviews [13].

The following research questions were asked to examine the recruitment methods used to actively involve people with a Turkish migration background living with dementia and their (informal) caregiving relatives in dementia care research:

1. What are the applied recruitment strategies for Turkish immigrants living with dementia and their caregiving relatives?
2. What are the facilitators unique to these recruitment strategies?
3. What are the barriers unique to these recruitment strategies?

### Inclusion and exclusion criteria

The eligibility criteria, as shown in Table 1, were determined using the PCC model (P = population, C = Concept, C = Context). The population for this review was Turkish immigrants with dementia living in countries other than their home country. The concept component was fulfilled by a focus on recruitment strategies to examine the methods used to recruit the target population in the studies. The context component was fulfilled by the inclusion of dementia care studies. Articles written in English or German and published in peer-reviewed journals were included.

**Table 1:** Inclusion and exclusion criteria for selecting studies

Inclusion criteria	Exclusion criteria
Focus on Turkish immigrants living with dementia	Not aligned with the intended target group (i.e., nonmigration background, not living with dementia, not being a caregiving relative)
Focus on individuals who are a caregiving relative, and who cares for a person living with dementia who is a Turkish immigrant	Professional caregivers
Information on recruitment strategies	
Peer-reviewed articles	
Articles written in English or German	
All types of studies	

### Types of sources

This scoping review included both experimental and quasi-experimental study designs, such as (cluster) randomized controlled trials, nonrandomized controlled trials, before–after studies, and interrupted time-series studies. In addition, descriptive and analytical observational studies, including prospective and retrospective cohort studies, case studies and analytical cross-sectional studies, were considered for inclusion. Qualitative studies such as phenomenology, grounded theory, ethnography, qualitative description, action research, and feminist and intersectionality research were also considered for inclusion.

### Search strategy

The search strings were adjusted for the databases MEDLINE/PubMed, PsycINFO, CINAHL, Scopus, Ovid Medline and Epub to identify studies published in the English language. Through the local databases of University Bielefeld, University Duisburg-Essen and Scopus, we identified articles published in the German language. The search terms included recognized Medical Subject Headings (MeSH), Titles (Ti) and Abstracts (Ab), and the search was conducted with the search terms shown in Supplement 1. The studies were identified by searching in two languages (English and German), and only literature published in peer-reviewed journals was selected. No restrictions were set regarding the publication date or the study type The search took place from April 28th - 30th, 2025, and screening was conducted by EI and SG. The study list was obtained by analysing articles from the reference lists of the included articles and by a manual search.

### Study selection

All identified articles were collated and uploaded into Endnote Version 20.5, and duplicates were removed. Following a pilot test, titles and abstracts were screened by two independent reviewers (EI, SG) against the inclusion criteria for this review. Potentially relevant sources were retrieved for full-text screening and assessed in detail against the inclusion criteria by two independent reviewers (EI, SG). Reasons for exclusion of the full text were discussed among EI and SG, any disagreements between the two reviewers at each stage of the selection process were resolved through discussion, and if no consensus was reached, a disagreement was resolved through discussion with the last author (MR). The results of this process are reported in the scoping review and presented in a Preferred Reporting Items for Systematic Reviews and Meta-analyses extension for scoping review (PRISMA-ScR) flow diagram [14].

**Fig. 1:**
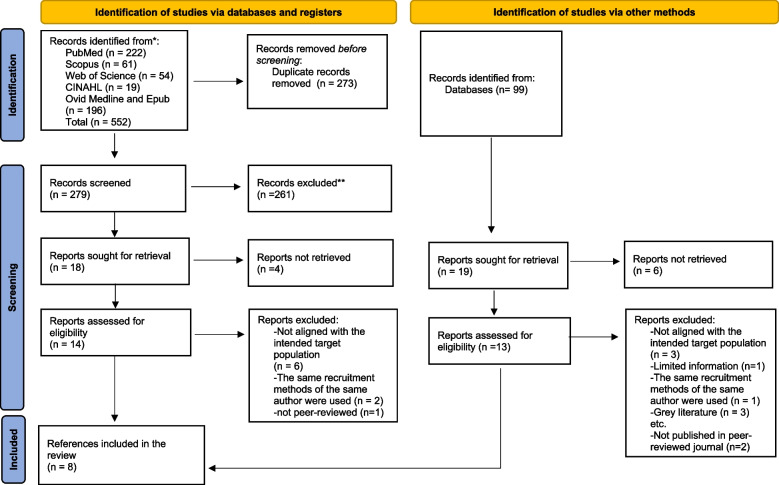
PRISMA-ScR Flow diagram. *Consider, if feasible to do so, reporting the number of records identified from each database or registry searched (rather than the total number across all databases/registries). **If automation tools were used, indicate how many records were excluded by a human and how many were excluded by automation tools. *From:* Page MJ, McKenzie JE, Bossuyt PM, Boutron I, Hoffmann TC, Mulrow CD, et al. The PRISMA 2020 statement: an updated guideline for reporting systematic reviews. BMJ 2021;372:n71. 10.1136/bmj.n71. For more information, visit: http://www.prisma-statement.org/

A total of 288 of 651 articles were identified after removing duplicates. Among 288 of the identified studies with different search strategies, 332 were excluded during the screening of titles and abstracts, and 29 were excluded during the full-text screening. As a result, five articles published in the English language and three articles published in the German language (combined *n* = 8) were considered eligible for the review. In addition, the reference lists of these selected articles were manually searched for additional articles, and three relevant articles were identified. Furthermore, we searched Google Scholar, and four more references were included. Thus, the final sample consisted of 15 articles for data extraction and analysis of facilitators and barriers **(**Fig. 1**).** Data were extracted for the following variables: year of publication, country in which the study was conducted, study type, sample size of people living with dementia (PLWD), study setting, description of the recruitment strategy, results with a focus on recruitment, and the key take-away message (Supplement 2 and Supplement 3).

## Results

In exploring the applied recruitment strategies for Turkish immigrants living with dementia and their caregiving relatives, this study delves into the distinct facilitators and barriers that shape the effectiveness of these recruitment approaches. Six of the fifteen articles were preclinical [7, 15–19]. Six studies were focused on PLWD [7, 15–19], and the others were focused on caregiver relatives [5, 9–11, 20–24]. Of the 15 studies, six studies were conducted in the Netherlands, five in Germany, three in Denmark, and one in Belgium. The studies published in the German language were primarily focused on the caregiving relatives of individuals with dementia rather than the individuals themselves. The review indicated that the majority of the studies related to Turkish immigrants and dementia are conducted by the same group of authors, depending on the country where the survey is being conducted.

Mostly, people of other ethnic groups were recruited together with Turkish immigrants in the included studies. In particular, Moroccan (*n* = 6), Surinamese (*n* = 4), Pakistani and Arabic-speaking (*n* = 3) immigrants were the focus. Additionally, immigrants from Italy and immigrants from Iran, Yugoslavia, Cape Verde, Iraq, Afghanistan, Palestina, Syria, Egypt, China, Venezuela, and Macedonia (*n* = 1) were recruited. In some articles, recruitment strategies were compared (immigrants versus non-immigrants, *n* = 4). The data extraction tables for PLWD and their caregiving relatives can be found in Supplement 2 and Supplement 3, respectively.

### Characteristics of the implemented recruitment strategies for PLWD and caregiving relatives

Content analysis was performed to identify the characteristics of the most implemented and effective strategies. The following five types of recruitment strategies were identified: healthcare environment outreach, inclusive practices and cultural considerations, face-to-face interactions, technology-mediated outreach, and engagement events.

- Healthcare Environments Outreach recruitment strategies consist of recruitment from institutions or care services providing medical treatment for PLWD. This category comprises geriatric/medical clinics, retirement homes, and home care.
- Inclusive Practices and Cultural Considerations recruitment strategies consider the needs and preferences of the target group and includes recruitment strategies that involve the use of bilingual materials (which allows participants to choose their preferred language), the employment of bilingual staff, and the education of staff/facilitators with basic theoretical training in dementia and culturally sensitive topics such as "information on dementia in relation to minority ethnic communities" [18].
- Face-to-Face Interactions recruitment strategies include the use social network strategies such as snowball sampling through stakeholders [5]. Professional and personal networks are considered [9]. In addition to social networks, physician referrals are also considered part of the face-to-face interactions category.
- Technology-Mediated Outreach recruitment strategies involve the use of registries/databases that provide information on individuals affected by dementia, including government-based and health organization-based registry systems, such as the Psychiatric Central Research Registry. Additionally, digital advertisements, phone calls, and mail/posts are considered in this category.
- Engagement Events recruitment strategies include organized special events, i.e., ethnic, religious, health-related, and association events, and they provide information on dementia. These events gather the target population in one place and make it easier to reach potential participants (or facilitators) who meet the inclusion criteria.

The recruitment strategies are summarized in four tables. The strategies for recruiting PLWD are shown in Table 2, those for recruiting their caregiving relatives are shown in Table 3, and those for recruiting facilitators are shown Tables 4 and 5; in addition, the barriers to recruitment are summarized in Tables 6 and 7.

**Table 2:** Recruitment strategies for Turkish immigrants living with dementia

		Included articles					
**Types of Recruitment**	**Recruitment strategy**	Celik et al. (2021)	Franzen et al. (2019)	Goudshmit et al. (2017)	Nielsen et al. (2022)	Nielsen et al. (2012)	Parlevliet et al. (2016)
Healthcare Environments Outreach	Health care/ Community organization					X	X
	Senior centres/Medical clinics/Home care provider	X	X	X			
Inclusive Practices and Cultural Considerations	Bilingual material					X	X
	Bilingual staff			X		X	X
	Education of staff				X		
Face-to-Face Interactions	Referral by physicians	X		X			X
Technology-Mediated Outreach	Database/Registry	X				X	
	Paid advertisement/ Social Media				X	X	X
	Phone calls/Email					X	X
Engagement Events	Professionals (bilingual) events				X

**Table 3:** Recruitment strategies for caregiving relatives

		Included articles								
Types of Recruitment	Recruitment strategy	Claeys et al. (2025)	Mogar & von Kutzleben (2015)	Monsees et al. (2020)	Nielsen et al. (2020)	Piechotta & Matter (2008)	Tezcan-Güntekin (2018)	Van der Heide et al. (2021)	Van Wezel et al. (2016)	Van Wezel et al. (2022)
Healthcare Environments Outreach	Health care/Community organization		X	X	X	X		X	X	X
	Senior centres/Medical clinics/Home care provider		X		X	X	X	X	X	
Inclusive Practices and Cultural Considerations	Bilingual material	X					X	X		X
	Bilingual staff	X					X		X	X
	Religious institutions							X		X
Face-to-Face Interactions	Social networks	X	X	X	X	X				
Technology-Mediated Outreach	Database/Registry					X				
	Phone calls/Email	X	X			X				
Engagement Events	Professionals (bilingual) events							X

**Table 4:** Facilitators identified for people of Turkish immigrants living with dementia

Recruitment strategy	Celik et al. (2021)	Franzen et al. (2019)	Goudsmit et al. (2017)	Nielsen et al. (2012)	Nielsen et al. (2022)	Parlevliet et al. (2016)	Stevensborg et al. (2016)
	Facilitating factors for successful recruitment						
Community nurse					X		
Bilingual research assistant				X			
Referral by a physician	X

**Table 5:** Facilitators identified for caregiving relatives of people of Turkish immigrants living with dementia

Recruitment strategy	Claeys et al. (2025)	Mogar & von Kutzleben (2015)	Monsees et al. (2020)	Nielsen et al. (2021)	Piechotta & Matter (2008)	Tezcan-Güntekin (2018)	Van der Heide et al. (2021)	Van Wezel et al. (2016)	Van Wezel et al. (2022)
	Facilitating factors for successful recruitment								
Healthcare professionals	X	X	X	X	X	X	X	X	X
Community partners	X	X	X	X					X
Healthcare organizations		X		X	X		X		X
Religious leaders							X		X
Key individuals				X		X		X	X

**Table 6:** Barriers identified for people of Turkish immigrants living with dementia

Included articles	Barriers to successful recruitment						
	Celik et al. (2021)	Franzen et al. (2019)	Goudsmit et al. (2017)	Nielsen et al. (2012)	Nielsen et al. (2022)	Parlevliet et al. (2016)	Stevensborg et al. (2016)
Language of the recruited population		X			X		
Cultural sensitivity and values	X				X		
Fear of being identified/stigmatized						X

**Table 7:** Barriers identified for caregiving relatives of people of Turkish immigrants living with dementia

Included articles	Barriers to successful recruitment								
	Claeys et al. (2025)	Mogar & von Kutzleben (2015)	Monsees et al. (2020)	Nielsen et al. (2021)	Piechotta & Matter (2008)	Tezcan-Güntekin (2018)	Van der Heide et al. (2021)	Van Wezel et al. (2016)	Van Wezel et al. (2022)
Mismatch language		X		X	X				
Cultural sensitivity missing		X	X	X	X	X			
Mistrust						X			
Lack of knowledge about dementia				X	X	X			
Differences in perception of dementia				X	X	X			
Lack of information about services	X		X	X	X				
Lack of knowledge in politics & science					X				
Independence of PLWD				X					

Based on the identified articles, multiple strategies were sometimes employed to effectively reach the target population. Therefore, we categorized the strategies used in the included articles according to the type of strategy. For instance, if in one study people were recruited through medical clinics and retirement homes, this study was mentioned once for the recruitment type “Healthcare Environments” through three health-care organizations, once for the recruitment type “setting based”, and, if in the same study social networks were also used, then this study additionally was listed for the “Face-to face interactions” strategy. Mostly, different strategies were used simultaneously, which made it difficult sometimes to distinguish them clearly from each other (for more details of the included studies, see Supplement 2).

### Frequency of identified recruitment strategies

Different approaches were observed in the strategies used for PLWD compared to their caregiving relatives. Six studies involved the recruitment of PLWD patients, and nine involved the recruitment of caregiving relatives. The Healthcare Environments Outreach category was frequently used for both target groups, especially for caregiving relatives. Caregiving relatives were primarily reached through elderly day care/outpatient care services (*n* = 4), nursing/care homes (*n* = 2), and hospitals (*n* = 3). For PLWD, hospitals/medical centres were preferred for this category (*n* = 3).

The inclusive practices and cultural considerations category include interventions that consider the needs and preferences of the target group. This recruitment strategy category was used slightly more frequently for caregiving relatives (*n* = 5) than for PLWD (*n* = 4). In contrast to the population of caregiving relatives, in one study, education regarding culturally sensitive topics in the recruitment of PLWD was provided for staff. The use of bilingual staff and materials was applied to both groups at nearly the same frequency, with caregiving relatives (*n* = 5) and PLWD (*n* = 4).

Face-to-face strategies included recruitment through social networks and referral by physicians. The use of social networks (*n* = 5) was only reported for recruiting caregiving relatives, and the snowball method was implemented in two of those studies. On the other hand, referrals by physicians were reported in three studies for PLWD and in only one study for caregiving relatives.

Regarding PLWD, the recruitment type Technology-Mediated Outreach was often preferred. These methods usually involved the use of registries and databases (*n* = 2). These registries were sourced from hospitals (*n* = 1) and from the Danish civil registration system (DCRS) (*n* = 1). The potential participants were mostly contacted via phone in two articles, while in one article, information letters were sent to potential participants. Regarding the recruitment of caregiving relatives, only one article mentioned a registry-based intervention, and it involved obtaining information from dementia/Alzheimer's associations. For this target group, information was also sent via mail, but to a lesser extent (*n* = 1). Recruitment through engagement events is mentioned the least. In one article, a dementia program was organized to provide information for ethnic minorities [18] and another, especially for people who are born in Turkey or Morocco [22].

Apart from the categories, the most frequently mentioned strategy is recruitment through organizations, which do not provide any medical treatment for individuals and differ from the Healthcare Environments Outreach. Based on the available studies, interventions implemented through health-related, community, or government-based organizations (*n* = 11) showed the highest success rates. The majority of caregiving relatives were recruited through these organizations (*n* = 8), which were primarily dementia-related organizations (*n* = 3), community organizations (*n* = 4) or both (*n* = 1), while recruitment through religious organizations/establishments (*n* = 2) was not implemented for PLWD. Mostly, facilitators (*n* = 6) from these various organizations were the individuals who contacted the target group.

### Characteristics of identified facilitators

Facilitators play a crucial role in recruiting caregiving relatives; interestingly, facilitators of PLWD were mentioned in only three studies. In every study that focused on caregiving relatives, facilitators could be identified. Most caregiving relatives in the studies were recruited through healthcare organizations (*n* = 5). In five of nine studies, the involvement of Alzheimer Associations [5, 11, 20, 22, 24] or community organizations was utilized to successfully recruit potential participants. Other facilitators for successfully recruiting caregiving relatives included professionals (*n* = 9), such as physicians (*n* = 2), nurses (*n* = 3), staff members in elderly day care (*n* = 3), and case managers (*n* = 2). In four out of nine studies, the involvement of key individuals from the community or (cultural) link workers (who have “professional or personal experience with minority ethnic older PLWD; and being of Turkish, Pakistani or Arabic speaking background” [9]) without depending on any institution facilitated successful recruitment. Furthermore, facilitators were linked to the involvement of care services (*n* = 5), which could be divided into four sections: (1) immigrant/ethnic-minority care organizations, which specialize in the target group (*n* = 3); (2) support groups for caregiving relatives of Turkish immigrants (*n* = 1); and (3) outpatient care services or hospitals (*n* = 2). Interestingly, religious leaders (such as imams from mosques) only partly functioned as facilitators (*n* = 2). Overall, we assumed that people who are trusted by potential participants (target group) are individuals, especially the target group, who share the same language/heritage/religion as the target population.

### Characteristics of the identified barriers

There were different types of barriers to the recruitment of Turkish immigrants living with dementia. There were more barriers reported for recruiting caregiving relatives than for recruiting PLWD. The main barriers were cultural topics (*n* = 5) and language mismatches, such as conducting interviews only in the language of the country of immigration [20] (*n* = 3). On the other hand, cultural differences in the perception of dementia, a lack of knowledge about the disease, a lack of information about available services and being unfamiliar with the health system (*n* = 4) were also barriers to participation in dementia studies. Mistrust of physicians was also one of the identified barriers (*n* = 1). In contrast to PLWD, a lack of knowledge in politics and science was identified as a barrier to becoming involved. In addition to the identified barriers for the caregiving relatives, there was a fear of being identified as a PLWD and thus being stigmatized (*n* = 1). PLWD do not want to give up on their personality and want to continue to participate in their regular life, but being involved in a study about dementia, being labelled a person with a disability, is linked with the fear of being stigmatized and the fear of being discriminated against when in need of treatment.

## Discussion

In this review, the authors aim to investigate the recruitment strategies employed for Turkish immigrants living with dementia and their caregiving relatives, along with the associated facilitators and barriers. The scoping review identified several recruitment strategies applied in engaging Turkish immigrants in dementia care research, which were categorized into five main strategies: Healthcare Environments Outreach, Inclusive Practices and Cultural Considerations, Face-to-Face interactions, Technology-Mediated Outreach, and Engagement Events. The Inclusive Practices and Cultural Considerations and Face-to-Face Interactions recruitment strategies were predominantly used for both target groups (PLWD and caregiving relatives). Cultural-sensitive strategies encompassed language-sensitive materials, offering the choice of using the preferred language for study tasks (e.g., conducting interviews), meetings at preferred locations, and researchers representing the same cultural background as the target group.

PLWD and their caregiving relatives were often recruited through key stakeholders, remote methods (such as databases, emails, or phone calls), or professionals (GPs or clinical staff). Step-by-step and snowballing approaches, combining different recruitment strategies, were used. The review revealed a lack of critical evaluation or adjustments to recruitment strategies during the study, with limited comments on their success. Similar clustering of recruitment strategies was found in a review by Gilmore-Bykovskyi, Jin [1] which emphasized local community centres in communities with high ethnic [1] minority populations as access points.

Despite numerous activities and culturally sensitive recruitment strategies, low response rates were reported in the included studies. The themes identified in other studies for ethnic minority groups without a focus on heritage included cultural perceptions of dementia, illness and older people; the impact of cultural perceptions on service use; and cross-cultural communication [25]. Minority group face barriers to get included in dementia care research, which varies based on their heritage. Similar to Turkish individuals, cultural values and differences in the perception of dementia play a crucial role for Korean Americans [26] and Chinese Americans [27]. While cultural sensitivity plays a crucial role for recruitment of Turkish immigrants, mistrust revealed as a less relevant impediment for Turkish immigrants in contrast to African Americans. This ethnic group faces barriers such as the lack of trust towards researchers and the medical system, language barriers, and financial limitations.

While these strategies mirror those used with other ethnic minority populations, their implications must be interpreted within the specific context of the Turkish diaspora in Europe. This population, especially in countries like Germany, the Netherlands, and Belgium, consists of multiple generations with diverse levels of acculturation. Many older adults migrated in the 1960s–1980 s as labor migrants and have limited proficiency in the host country's language and formal education. This linguistic and educational background amplifies the need for culturally and linguistically sensitive recruitment approaches. Turkish communities in European countries are often characterized by strong intra-community networks—including religious organizations, ethnic associations, and Turkish-language media—which can serve as crucial entry points for recruitment. Moreover, the heterogeneity within the Turkish diaspora—in terms of migration history, socioeconomic status, and integration—means that one-size-fits-all strategies are unlikely to be effective.

Several key barriers to research participation emerged in this review, particularly language limitations, cultural differences in understanding dementia, and stigma. These barriers are deeply rooted in the historical, social, and structural experiences of Turkish immigrants in Europe. Many older Turkish adults have limited proficiency in the host country’s language, and health materials are rarely available in Turkish or adapted to low literacy levels. Furthermore, dementia is often perceived not as a medical condition but as a natural part of aging or even as a moral or spiritual issue, which can contribute to stigma and denial. These perceptions hinder not only help-seeking but also openness to research participation, particularly when the purpose of the research is not clearly explained in a culturally resonant manner. To mitigate these barriers, researchers must move beyond simple translation of materials. Culturally appropriate recruitment strategies should include collaboration with community leaders, use of trusted institutions (e.g., mosques, Turkish cultural centers), and researcher reflexivity about their own positionality. Recruitment messages should reflect culturally relevant values such as family responsibility, honor, and respect for elders, and may be more effective if framed in terms of contributing to the well-being of the community, rather than as individual participation. Future studies should systematically incorporate and evaluate community co-leadership and participatory recruitment models, which could foster mutual trust and sustained engagement.

The limited reporting on the effectiveness or adaptability of recruitment strategies also points to a missed opportunity. Very few studies critically evaluated their recruitment process or adapted strategies mid-course. This is particularly important for Turkish-origin populations, where stepwise engagement and trust-building may require multiple contacts, as shown in longitudinal research from Germany [28].

Recruitment success depended on the establishment of community partnerships and the addressing of individuals' suspicions. Employing a venue-based sampling method, a tailored communication strategy, and the snowball sampling method played a pivotal role [29]. Connecting participants with relevant resources facilitated effective and non-instrumental engagement. The formation of a diverse, multicultural team proved beneficial for building trust [29]. Previous research highlights the importance of employing culturally tailored and inclusive recruitment strategies when engaging migrant populations in health studies. Face-to-face interactions often emerge as more effective than digital methods, particularly for communities with limited language proficiency or digital access. These studies underscore the importance of expanding the research evidence base to better address the needs and perspectives of minority populations, particularly those disproportionately affected by disease burdens [30]. One study investigated potential distinctions in participant characteristics between community-based sampling (CBS) and registry-based sampling (RBS) approaches for individuals of Turkish origin in three German cities. The CBS approach led to an enhanced representation of individuals of Turkish origin with lower language skills and acculturation status [31]. In the first cohort study in Germany, which examined retention and possible strategies as well as differences between participants and nonparticipants among people of Turkish origin, it was shown that each successive level of contact significantly increased the retention rate among study participants of Turkish origin. Therefore, investing in comprehensive retention strategies that take cultural characteristics into account will lead to higher validity and greater statistical power in cohort studies of migrants [28].

## Limitations

The studies included in this scoping review did not specifically delve into the analysis or evaluation of participants’ own recruitment strategies. Consequently, our search focused on extracting relevant information from the identified studies that involved individuals of Turkish origin or Turkish migrants and their care-giving relatives participating in dementia care studies. Another limitation arises from the inclusion criterion, as only studies in English and German languages were considered, excluding those in Turkish. Initial efforts involved a manual search for Turkish-language studies, but none were found to meet the inclusion criteria. Consequently, the decision to concentrate on articles in English and German was deemed appropriate. The third constraint is associated with the geographical distribution of the studies. Specifically, five studies were carried out in Germany, two studies were conducted in Denmark, and three studies took place in the Netherlands. These findings collectively represent the outcomes from the European region.

## Conclusion

In conclusion, this scoping review elucidates various recruitment strategies employed in dementia care research focusing on Turkish immigrants. The findings highlight the diverse approaches undertaken to engage this specific population in research endeavours. We conclude that the available evidence of successful recruitment strategies to include underrepresented populations in dementia care research is limited. Despite the variations in recruitment methods, this review underscores the importance of considering cultural nuances, language proficiency, and acculturation levels when designing recruitment strategies for Turkish immigrants in dementia care research. As the field continues to evolve, this synthesis of recruitment approaches provides valuable insights for researchers and practitioners seeking effective and culturally sensitive methods to enhance participant inclusion and representation in studies focused on dementia care within the Turkish immigrant community. We can successfully create opportunities for engagement, but there is no 'one size fits all' recruitment strategy. The comprehensive overview presented in this scoping review serves as a foundation for future research endeavours aiming to advance the understanding of dementia care among Turkish immigrants and to further refine recruitment strategies tailored to the unique needs of this population.

## Supplementary Information

Supplementary Material 1.

Supplementary Material 2.

Supplementary Material 3.

Supplementary Material 4.

## Data Availability

No datasets were generated or analysed during the current study.

## Abbreviations

PLWD: People Living with Dementia
PRISMA-ScR: The PRISMA Scoping Reviews Extension
