# Supplementary material for: Recruitment strategies for Turkish immigrants in dementia care research: a scoping review
Source: BMC Geriatr. 2025 Jun 5;25:411. doi: 10.1186/s12877-025-06031-3 (PMC12139117; doi:10.1186/s12877-025-06031-3)
Supplement: Supplementary file 2 — Supplementary Material 2. [file 12877_2025_6031_MOESM2_ESM.docx]

**Pubmed**

5 ((("Cognitive Dysfunction"[Mesh] OR "Alzheimer Disease"[Mesh] OR "Alzheimer Disease" OR Dementia OR Alzheimer OR "Cognitive Disorder" OR "Cognitive Dysfunction" OR "Cognitive Impairment" OR "Cognitive Decline" OR "Mental Deterioration") AND (Turkish OR Turk*)) AND ("Emigrants and Immigrants"[Mesh] OR immigrant OR foreigner OR emigrant OR minority OR minorities OR origin OR background)) AND (Recruit* OR Outreach* OR Engag* OR Enroll* OR Retention* OR Inclusion* OR Participat* OR "recruitment strategy" OR recruitment strateg*) Publication Date ("Cognitive Dysfunction"[MeSH Terms] OR "Alzheimer Disease"[MeSH Terms] OR "Alzheimer Disease"[All Fields] OR ("dementia"[MeSH Terms] OR "dementia"[All Fields] OR "dementias"[All Fields] OR "dementia s"[All Fields]) OR ("alzheime s"[All Fields] OR "Alzheimer Disease"[MeSH Terms] OR ("alzheimer"[All Fields] AND "disease"[All Fields]) OR "Alzheimer Disease"[All Fields] OR "alzheimer"[All Fields] OR "alzheimers"[All Fields] OR "alzheimer s"[All Fields] OR "alzheimers s"[All Fields]) OR "Cognitive Disorder"[All Fields] OR "Cognitive Dysfunction"[All Fields] OR "Cognitive Impairment"[All Fields] OR "Cognitive Decline"[All Fields] OR "Mental Deterioration"[All Fields]) AND ("Turkish"[All Fields] OR "turk*"[All Fields]) AND ("Emigrants and Immigrants"[MeSH Terms] OR ("Emigrants and Immigrants"[MeSH Terms] OR ("emigrants"[All Fields] AND "immigrants"[All Fields]) OR "Emigrants and Immigrants"[All Fields] OR "immigrant"[All Fields] OR "immigrants"[All Fields] OR "emigration and immigration"[MeSH Terms] OR ("emigration"[All Fields] AND "immigration"[All Fields]) OR "emigration and immigration"[All Fields] OR "immigration"[All Fields] OR "immigrations"[All Fields] OR "immigrant s"[All Fields] OR "immigrate"[All Fields] OR "immigrated"[All Fields] OR "immigrates"[All Fields] OR "immigrating"[All Fields]) OR ("Emigrants and Immigrants"[MeSH Terms] OR ("emigrants"[All Fields] AND "immigrants"[All Fields]) OR "Emigrants and Immigrants"[All Fields] OR "foreigner"[All Fields] OR "foreigners"[All Fields]) OR ("Emigrants and Immigrants"[MeSH Terms] OR ("emigrants"[All Fields] AND "immigrants"[All Fields]) OR "Emigrants and Immigrants"[All Fields] OR "emigrant"[All Fields] OR "emigrants"[All Fields] OR "emigrate"[All Fields] OR "emigrated"[All Fields] OR "emigrates"[All Fields] OR "emigrating"[All Fields] OR "emigration and immigration"[MeSH Terms] OR ("emigration"[All Fields] AND "immigration"[All Fields]) OR "emigration and immigration"[All Fields] OR "emigration"[All Fields] OR "emigrations"[All Fields] OR "emigres"[All Fields]) OR ("minority groups"[MeSH Terms] OR ("minority"[All Fields] AND "groups"[All Fields]) OR "minority groups"[All Fields] OR "minorities"[All Fields] OR "minority"[All Fields] OR "minority s"[All Fields] OR "minors"[MeSH Terms] OR "minors"[All Fields] OR "minor"[All Fields]) OR ("minority groups"[MeSH Terms] OR ("minority"[All Fields] AND "groups"[All Fields]) OR "minority groups"[All Fields] OR "minorities"[All Fields] OR "minority"[All Fields] OR "minority s"[All Fields] OR "minors"[MeSH Terms] OR "minors"[All Fields] OR "minor"[All Fields]) OR ("origin"[All Fields] OR "originate"[All Fields] OR "originated"[All Fields] OR "originates"[All Fields] OR "originating"[All Fields] OR "origination"[All Fields] OR "originations"[All Fields] OR "origins"[All Fields]) OR ("background"[All Fields] OR "backgrounds"[All Fields])) AND ("recruit*"[All Fields] OR "outreach*"[All Fields] OR "engag*"[All Fields] OR "enroll*"[All Fields] OR "retention*"[All Fields] OR "inclusion*"[All Fields] OR "participat*"[All Fields] OR "recruitment strategy"[All Fields] OR (("recruit"[All Fields] OR "recruited"[All Fields] OR "recruiter"[All Fields] OR "recruiters"[All Fields] OR "recruiting"[All Fields] OR "recruitment"[All Fields] OR "recruitments"[All Fields] OR "recruits"[All Fields]) AND "strateg*"[All Fields])) 156

4 Recruit* OR Outreach* OR Engag* OR Enroll* OR Retention* OR Inclusion* OR Participat* OR "recruitment strategy" OR recruitment strateg* Publication Date "recruit*"[All Fields] OR "outreach*"[All Fields] OR "engag*"[All Fields] OR "enroll*"[All Fields] OR "retention*"[All Fields] OR "inclusion*"[All Fields] OR "participat*"[All Fields] OR "recruitment strategy"[All Fields] OR (("recruit"[All Fields] OR "recruited"[All Fields] OR "recruiter"[All Fields] OR "recruiters"[All Fields] OR "recruiting"[All Fields] OR "recruitment"[All Fields] OR "recruitments"[All Fields] OR "recruits"[All Fields]) AND "strateg*"[All Fields]) 2,369,127

3 "Emigrants and Immigrants"[Mesh] OR immigrant OR foreigner OR emigrant OR minority OR minorities OR origin OR background Publication Date "Emigrants and Immigrants"[MeSH Terms] OR ("Emigrants and Immigrants"[MeSH Terms] OR ("emigrants"[All Fields] AND "immigrants"[All Fields]) OR "Emigrants and Immigrants"[All Fields] OR "immigrant"[All Fields] OR "immigrants"[All Fields] OR "emigration and immigration"[MeSH Terms] OR ("emigration"[All Fields] AND "immigration"[All Fields]) OR "emigration and immigration"[All Fields] OR "immigration"[All Fields] OR "immigrations"[All Fields] OR "immigrant s"[All Fields] OR "immigrate"[All Fields] OR "immigrated"[All Fields] OR "immigrates"[All Fields] OR "immigrating"[All Fields]) OR ("Emigrants and Immigrants"[MeSH Terms] OR ("emigrants"[All Fields] AND "immigrants"[All Fields]) OR "Emigrants and Immigrants"[All Fields] OR "foreigner"[All Fields] OR "foreigners"[All Fields]) OR ("Emigrants and Immigrants"[MeSH Terms] OR ("emigrants"[All Fields] AND "immigrants"[All Fields]) OR "Emigrants and Immigrants"[All Fields] OR "emigrant"[All Fields] OR "emigrants"[All Fields] OR "emigrate"[All Fields] OR "emigrated"[All Fields] OR "emigrates"[All Fields] OR "emigrating"[All Fields] OR "emigration and immigration"[MeSH Terms] OR ("emigration"[All Fields] AND "immigration"[All Fields]) OR "emigration and immigration"[All Fields] OR "emigration"[All Fields] OR "emigrations"[All Fields] OR "emigres"[All Fields]) OR ("minority groups"[MeSH Terms] OR ("minority"[All Fields] AND "groups"[All Fields]) OR "minority groups"[All Fields] OR "minorities"[All Fields] OR "minority"[All Fields] OR "minority s"[All Fields] OR "minors"[MeSH Terms] OR "minors"[All Fields] OR "minor"[All Fields]) OR ("minority groups"[MeSH Terms] OR ("minority"[All Fields] AND "groups"[All Fields]) OR "minority groups"[All Fields] OR "minorities"[All Fields] OR "minority"[All Fields] OR "minority s"[All Fields] OR "minors"[MeSH Terms] OR "minors"[All Fields] OR "minor"[All Fields]) OR ("origin"[All Fields] OR "originate"[All Fields] OR "originated"[All Fields] OR "originates"[All Fields] OR "originating"[All Fields] OR "origination"[All Fields] OR "originations"[All Fields] OR "origins"[All Fields]) OR ("background"[All Fields] OR "backgrounds"[All Fields]) 4,020,949

2 Turkish OR Turk* Publication Date "Turkish"[All Fields] OR "turk*"[All Fields] 410,593

1 "Cognitive Dysfunction"[Mesh] OR "Alzheimer Disease"[Mesh] OR "Alzheimer Disease" OR Dementia OR Alzheimer OR "Cognitive Disorder" OR "Cognitive Dysfunction" OR "Cognitive Impairment" OR "Cognitive Decline" OR "Mental Deterioration" Publication Date "Cognitive Dysfunction"[MeSH Terms] OR "Alzheimer Disease"[MeSH Terms] OR "Alzheimer Disease"[All Fields] OR ("dementia"[MeSH Terms] OR "dementia"[All Fields] OR "dementias"[All Fields] OR "dementia s"[All Fields]) OR ("alzheime s"[All Fields] OR "Alzheimer Disease"[MeSH Terms] OR ("alzheimer"[All Fields] AND "disease"[All Fields]) OR "Alzheimer Disease"[All Fields] OR "alzheimer"[All Fields] OR "alzheimers"[All Fields] OR "alzheimer s"[All Fields] OR "alzheimers s"[All Fields]) OR "Cognitive Disorder"[All Fields] OR "Cognitive Dysfunction"[All Fields] OR "Cognitive Impairment"[All Fields] OR "Cognitive Decline"[All Fields] OR "Mental Deterioration"[All Fields] 402,730

**Scopus**

TITLE-ABS-KEY ("Alzheimer Disease" OR dementia OR alzheimer OR "Cognitive Disorder" OR "Cognitive Dysfunction" OR "Cognitive Impairment" OR "Cognitive Decline" OR "Mental Deterioration" ) AND TITLE-ABS-KEY ( turkish OR turk* ) AND TITLE-ABS-KEY ( immigrant OR foreigner OR emigrant OR minority OR minorities OR origin OR background ) AND TITLE-ABS-KEY ( recruit* OR outreach* OR engag* OR enroll* OR retention* OR inclusion* OR participat* OR "recruitment strategy" )

48

**Web of Science**

1: TS=("Alzheimer Disease" OR dementia OR alzheimer OR "Cognitive Disorder" OR "Cognitive Dysfunction" OR "Cognitive Impairment" OR "Cognitive Decline" OR "Mental Deterioration" ) Date Run: Tue Jul 11 2023 15:06:48 GMT+0300 (GMT+03:00) Results: 435870

2: TS=(Turkish OR Turk*) Date Run: Tue Jul 11 2023 15:07:25 GMT+0300 (GMT+03:00) Results: 229455

3: TS=(immigrant OR foreigner OR emigrant OR minority OR minorities OR origin OR background) Date Run: Tue Jul 11 2023 15:07:53 GMT+0300 (GMT+03:00) Results: 4379669

4: TS=(recruit* OR outreach* OR engag* OR enroll* OR retention* OR inclusion* OR participat* OR "recruitment strategy") Date Run: Tue Jul 11 2023 15:08:29 GMT+0300 (GMT+03:00) Results: 3539078

5: #4 AND #3 AND #2 AND #1 Date Run: Tue Jul 11 2023 15:08:39 GMT+0300 (GMT+03:00) Results: 43

**CINAHL**

S3 S1 OR S2 Expanders - Also search within the full text of the articles; Apply equivalent subjects

Search modes - Boolean/Phrase Interface - EBSCOhost Research Databases

Search Screen - Advanced Search

Database - CINAHL Plus with Full Text 18

S2 TI ( "Alzheimer Disease" OR dementia OR alzheimer OR "Cognitive Disorder" OR "Cognitive Dysfunction" OR "Cognitive Impairment" OR "Cognitive Decline" OR "Mental Deterioration" ) AND TI ( Turkish OR Turk* ) AND TI ( immigrant OR foreigner OR emigrant OR minority OR minorities OR origin OR background ) AND TI ( recruit* OR outreach* OR engag* OR enroll* OR retention* OR inclusion* OR participat* OR "recruitment strategy" ) Expanders - Also search within the full text of the articles; Apply equivalent subjects

Search modes - Boolean/Phrase Interface - EBSCOhost Research Databases

Search Screen - Advanced Search

Database - CINAHL Plus with Full Text 0

S1 AB ( "Alzheimer Disease" OR dementia OR alzheimer OR "Cognitive Disorder" OR "Cognitive Dysfunction" OR "Cognitive Impairment" OR "Cognitive Decline" OR "Mental Deterioration" ) AND AB ( Turkish OR Turk* ) AND AB ( immigrant OR foreigner OR emigrant OR minority OR minorities OR origin OR background ) AND AB ( recruit* OR outreach* OR engag* OR enroll* OR retention* OR inclusion* OR participat* OR "recruitment strategy" ) Expanders - Also search within the full text of the articles; Apply equivalent subjects

Search modes - Boolean/Phrase Interface - EBSCOhost Research Databases

Search Screen - Advanced Search

Database - CINAHL Plus with Full Text 18

**Ovid MEDLINE(R) and Epub Ahead of Print, In-Process, In-Data-Review & Other Non-Indexed Citations, Daily and Versions <1946 to July 10, 2023>**

1 (("Alzheimer Disease" or dementia or alzheimer or "Cognitive Disorder" or "Cognitive Dysfunction" or "Cognitive Impairment" or "Cognitive Decline" or "Mental Deterioration") and (Turkish or Turk*) and (immigrant or foreigner or emigrant or minority or minorities or origin or background) and (recruit* or outreach* or engag* or enroll* or retention* or inclusion* or participat* or "recruitment strategy")).af. 138

**Total Records: 403**
