# Supplementary material for: Recruitment strategies for Turkish immigrants in dementia care research: a scoping review
Source: BMC Geriatr. 2025 Jun 5;25:411. doi: 10.1186/s12877-025-06031-3 (PMC12139117; doi:10.1186/s12877-025-06031-3)
Supplement: Supplementary file 3 — Supplementary Material 3. [file 12877_2025_6031_MOESM3_ESM.docx]

**Supplement 1: Data Extraction of the Recruitment Strategies for People with Dementia**

| **Author, year, country** | **Study Type** | **Aim** | **Sample size**  **Turks** | **Setting** | **Description of recruitment strategy** | | **Results through the lens of recruitment** | **Take away message** |
| --- | --- | --- | --- | --- | --- | --- | --- | --- |
| Goudsmit et al. (2017)  Netherlands | Psychometric Testing of the CCD  Part 1: Standardization  Part 2: Validity  (embedded in the larger study SYMBOL) | Examine the effect of ethnicity on the performance of the Cross-Cultural Dementia Screening (CCD) by comparing subtest scores in Dutch, Turkish, Moroccan-Berber, Moroccan-Arabic, and Surinamese-Creole and Surinamese-Hindustani groups. | N= 323 | Academic Medical Center,  GP's practice,  a local center,  or a home visit. | **Healthcare Environment Outreach:** Participants, who have probable dementia, were located in seven cities in the Netherlands with a large immigrant population. Patients were invited to join the study through a letter from their GP and the research team. | **Inclusive Practices and Cultural Considerations:** After they voiced interest in participation, a bilingual and bicultural interviewer explained the study via phone.  **Face-to-face Strategy**:  If they agreed to participate, an appointment was made (GP practice, local center or at home). | The different ethnic minority groups (based on self-defined ethnicity) showed differences in age, gender, and education.  Among 1625 participants, 323 of them were Turks. The average age of Turkish people was 63.6 (SD= 6.0) with a range of 55-83 years. 46% were male. | Bilingual/-cultural interviewers conducted the interviews/ questionnaires.  Recruitment requires awareness of illiteracy barriers, language barriers, and cultural differences.  Using the participants language and well-trained researchers is beneficiary.  Meeting in local community or specialized centers are suitable for (potential) participants. |
| Nielsen et al. (2022)  Denmark | Quasi-Experimental/ Pre-Post Test | Assess the feasibility of a culturally tailored dementia information program that provides basic knowledge about dementia to middle-aged and older people in minority ethnic communities (Turkish, Pakistani or Arabic-speaking heritage). | N = 65 | Community centers,  minority ethnic activity and social groups, participant's homes and social gatherings in collaboration and partnership with participating municipalities. | **Inclusive Practices and Cultural Considerations:** Participants were recruited from the communities through cultural link workers, minority ethnic groups, and social gatherings using advertisements, announcements in Danish and minority languages. | **Inclusive Practices and Cultural Considerations and Engagement Events:** Dementia information (program/) sessions were conducted at community centers, minority ethnic and social groups, at home | Most participants were middle-aged and older women with a general interest in age-related memory problems.  There was only one male participant in the program sessions. The low male participation was attributed to women's greater interest and activity in seeking health-related information. | Recruitment through established groups and word-of-mouth was more effective than distributing flyers or using community center announcements.  Providing mixed sessions (women and men) is difficult for participants. It is recommended to separately access the potential participants.  Not speaking the same language as the ethnic minority group acts as a barrier to reach the target group. |
| Nielsen et al. (2012)  Denmark | Psychometric Testing | Assess the performance of Turkish minority participants on the Rowland Universal Dementia Assessment Scale (RUDAS) and the Mini-Mental State Examination (MMSE) tests and to examine the impact of demographic and health-related characteristics on these measurements. | N= 76 | In their homes or in another location if they preferred. | **Technology-Mediated Outreach:**  The Danish Civil Registration System (DCRS) includes individual demographic data, such as date of immigration and country of residence before immigration). All Danish residents' interactions with the secondary healthcare system are recorded in two national medical registers. A random sample of 500 elderly Turkish minority individuals living in the community were contacted. | **Inclusive Practices and Cultural Considerations and Technology-Mediated Outreach:** The study employed bilingual research assistants, invitations letters were sent in Turkish and Danish. Follow-up phone calls, if the persons contacted did not respond to the invitation letter within 2 weeks.  **Face-to-face Strategy**: Potential participants were visited by a research neuropsychologist and interpreter at their home or another suitable location or at a hospital in Copenhagen. | 260 out of 500 originally identified individuals in the DCRS could not be reached as they were not listed in phonebooks.  Initially, 59 invitations were sent, and 32 agreed to participate.  Afterward, 185 more individuals were contacted through follow-up calls, with 51 accepting the study.  Among the 83 recruited participants, 59% were ethnic Kurds (N=49), 39% were ethnic Turks (N=32), and the remaining 2% had other ethnic backgrounds. Seven were excluded later. Participants were aged 50 and older. | Length of education influenced the results of the test (e.g. drawing required for the test).  Calendar system used by the older participants might be different then the system used by the new country.  Despite the fact that bilingual researchers were involved, material was provided in Turkish, and meetings were scheduled at a preferred location and all visits were planned outside the periods of summer holidays, Ramadan and Christmas participation rate was low. |
| Parlevliet et al. (2016)  Netherlands | Secondary Data Analysis of Data from a Cross-Sectional Study (SYMBOL) | Determining the prevalence of MCI and dementia in older community-dwelling adults from non-western immigrant groups in the Netherlands. | N = 540 | GP´s practice, social center, home of the participants. | **Face-to-face Strategy:** (Bilingual) invitations were sent by the GP´s in seven suburbs with large immigrant populations and by the research team.  **Inclusive Practices and Cultural Considerations with Technology-Mediated Outreach:** The research team got in contact through follow-up calls by trained bilingual and bicultural interviewers. Participants could choose their preferred language. | **Face-to-face Strategy**: The preferred location of the participants defined where meetings took place. | A total of 2254 participants were included in the study. The mean age was 65.0 years (SD= 7.5) with a range of 55 and older. 44.4% of them were male.  The study SYMBOL had a low participation rate (~31%) and different participation rates between ethnic groups.  Compared to non-responders, participants were younger and included fewer males. 41% of all non-responders were Moroccan. | The recruitment through the GP reached a high number of participants.  MCI and dementia prevalence was high among Turkish compared to native Dutch participants, which might be provide an argument for future recruitments.  Experiences such as shame might had influenced non-response.  People older than 85y could not be reached/motivated to participate. |
| Celik et al. (2021)^[[1]](#footnote-1)^  Germany | Cross-Cultural Comparison Study | Investigating the impact of culture, demographic, and immigration-related factors on the performance of two cognitive screening tools: the MMSE and RUDAS. | N= 24 monolingual Turkish speaking (living in Turkey)  N= 21 Turkish immigrants (living in Germany) | Central Institute of Mental Health Mannheim,  the Department of Neurology at the University of Cologne. | **Face-to-face Strategy and technology-Mediated Outreach:** Participants were referred by a neurologist or neurology clinic/ old age psychiatry service databases of hospitals | **Technology-Mediated Outreach**: Potential participants were contacted by the research team. If they were interested in the study a structured phone interview was conducted to learn more about their biography.  **Face-to-Face Strategy and Healthcare Environment Outreach**:  People eligible for the study were given an appointment at the participating hospital. | The average age of the participants was 71.65 years (SD = 7.41 years) and 43% were male. The age range was between 50 years and older.  If needed, a close family member helped (only relevant for participants in Germany) to answer some of the questionnaires.  Turkish immigrants living in Germany chose the Turkish version of the different measurements. | Cultural background (native Turks living in Turkey, Turkish immigrants vs. native German born) and education level (adjusted) had no independent effect on MMSE score. Variables associated with immigration experience also did not result in MMSE differences.  This information is relevant for researchers and preconceptions they may have when recruiting. |
| Franzen et al. (2019)  Netherlands | Psychometric Testing | Examine the impact of culture, demographic, and immigration-related factors on the performance of cognitive screening tools: the Mini-Mental State Examination (MMSE), the Cross Cultural Dementia Screening (CCD) and Rowland Universal Dementia Assessment Scale (RUDAS). | N= 34 with dementia diagnosis  N = 14 healthy Turkish immigrants | In outpatient multicultural memory clinics of the Erasmus MC University Medical Center; in former Havenziekenhuis in Rotterdam. | **Healthcare Environments Outreach:**  Non-Western immigrant patients were recruited from outpatient multi-cultural memory clinics at Erasmus MC University Medical Center and Havenziekenhuis in Rotterdam. | **Face-to-face Strategy**:  Interpreters were present during examinations and intake interview  Control group (healthy Turks) were assed in Turkish. | Regarding the 73 participants from the memory clinics with different origins, the average age was 68.48 years (SD = 11.00) and 55% were male.  The patients originated from various countries, including Turkey, Morocco, Cape Verde, Pakistan, Iraq, Afghanistan, and others.  Among 48 Turkish participants, for 14% of them dementia diagnosis could not be determined and or additional procedures failed or were refused. | The study revealed that the color of drawings (usually part of tests) had an impact on memory results.  To use color might be helpful for low-educated people, the ability to decode black-white line drawings is linked to education level and literacy training. These details are of relevance for the preparation of recruitment material and explanations of planned test in a study. |

1. **PS:** Information of the Recruitment of healthy Turkish elderly people is not included in the table [↑](#footnote-ref-1)
