# Supplementary material for: Recruitment strategies for Turkish immigrants in dementia care research: a scoping review
Source: BMC Geriatr. 2025 Jun 5;25:411. doi: 10.1186/s12877-025-06031-3 (PMC12139117; doi:10.1186/s12877-025-06031-3)
Supplement: Supplementary file 4 — Supplementary Material 4. [file 12877_2025_6031_MOESM4_ESM.docx]

**Supplement 2: Included Studies and Characteristics with Caregivers**

| **Author, year, country** | **Study Type** | **Aim** | **Sample (Turks)** | **Setting** | **Description of recruitment strategy** | | | **Results through the lens of recruitment** | **Take away message** |
| --- | --- | --- | --- | --- | --- | --- | --- | --- | --- |
| Claeys et al. (2025)  Belgium | In-depth Interviews | Describe experiences of informal caregivers of for older first-generation labour migrants from Italian and Turkish backgrounds with dementia | N= 8 | local community centre and local organisation | **Inclusive Practices and Cultural Considerations:**  Potential Participants were informed in various languages (Turkish, Dutch, French and Italian).  The interviews were conducted in Turkish, Dutch or French.  Bilingual staff were used. | | **Technology-mediated Outreach and Face-to-face Strategy:** Stakeholders within the local community and GP’s were contacted via phone to find potential participants. | Two of the eight Turkish caregivers were male. Five of the interviews were conducted in Dutch. Two participants required a Turkish interpreter, and one participant continued using both Dutch and a Turkish interpreter. Most of the eight caregiving relatives were daughters of the plwd (four), followed by two sons, one spouse, and one niece | Language preferences among informal caregivers varied by cultural background: while Italian-background caregivers tended to prefer French or Dutch, Turkish-background caregivers often required interpretation support. |
| Mogar M.  & von Kutzleben  (2015)  Germany | Semi-Structured Interviewa,  Exploratory Qualitative Study | Gain insight into the organization and characteristics of home-based care arrangements for people suffering from dementia with a Turkish migration background from a family carer´s perspective | N= 7 | Home care services,  support groups for caregiving relatives with Turkish migration background, local  Alzheimer Associations, Dementia-Service-Centers, Worker´s welfare associations and others. | **Face-to-face Strategy:** Two of the participants were recruited through personal contacts of the First-Author in her social environment. | **Face-to-face Strategy:** Interviews were conducted between 30 and 120 minutes. | | The PWLD who received home care were between 63-85 years and their caregiving relative between 24 and 50 years old. Out of the seven participants, four relatives were responsible for caring for their mothers, two for their grandmothers, and one for her mother-in-law. Two of the participants were people with a Kurdish background. All individuals with dementia were female, while only two of the caregivers were male. | Recruitment was very difficult. The primary caregiving relative is key for decision making processes.  Speaking the native language (Turkish or Kurdish) is of relevance to be able to reach the target group.  All interviews are conducted in German. |
| Monsees et al. (2020)  Germany | Qualitative Study, Semi-Structured Interviews | Examine the experience of family care-givers and to identify barriers of people with dementia and Turkish migration background using information and healthcare services | N= 8 | The German Alzheimer Assoziation, Dementia Support Stuttgart, Dementia Service Center Gelsenkirchen, “Landesverband der Alzheimer Gesellschaften NRW e. V.” | **Technology-mediated Outreach and Face-to-face Strategy:** Stakeholders in different dementia associations were contacted via phone or email to find potential participants. | **Face-to-face Strategy:** Participants were recruited through a snowball sampling method due to the difficulty in reaching them. Other individuals were reached through the social contacts of two participants. | | The age range of the relatives was between 38 and 56 years. Among the participants, only one is male. Four of the relatives provided care for their mother, two for their father, one for her mother and her father, and one for her grandmother. | The authors point out, that it was important to work with professionals of the same culture to contact the target population.  To use media (e.g., radio, TV, and newspaper) relevant to the target culture seems to be beneficiary  All interviews are conducted in German. |
| Nielsen et al. (2021)  Denmark | Semi-Structured Qualitative Individual and Group Interviews | Investigate obstacles in accessing dementia care among minority ethnic groups, including Turkish, Pakistani, and Arabic speakers. | N= 6 | In urban areas with large minority ethnic populations in Denmark (Odense and Copenhagen). | **Face-to-face Strategy:** Professional contacts from local dementia coordinators and snowballing to recruit minority ethnic family carers through dementia care coordinators, staff in elderly daycare. |  | | The age range of the family carers was between 38 and 70 and 8 of 12 participants (Turkish, Arabic & Pakistani) were female.  The family caregivers took care for a period range of 3-15 years for a family member with Alzheimer's disease. 2 of the relatives were spouses, 9 children (or children-in-law) and 1 grandchild. | Overall, access barriers are exaggerated by language and culture issues.  Awareness needed that perception of dementia may be different due to culture (family) values and therefore influences participation. Speaking Danish was not a requirement, but all participants spoke Danish. |
| Piechotta F. & C. Matter  2008  Germany | Survey,  Explorative Study | Investigate various perspectives and to provide concrete suggestions for a culturally sensitive approach to dementia-affected Turkish migrants and their caregiving relatives | N= 2 | Two Counseling centers for migrants; Two Medical Service of Health Insurance (MDK); Two hospitals; Two stationary interculturally oriented senior care facilities; Three inter-culturally oriented home care services and adult day care centres. | **Technology-mediated Outreach:**  Specialized address and phone lists from “Türkischer Bund Berlin” (TBB), Alzheimer-Association in Berlin and care providers to contact 40 institutions.  Information letters were sent out to these institutions and subsequently, telephone contact was established. | **Face-to-face strategy:** Two of the institutions referred two family members taking care for a person with dementia at home. | | Out of 40 institutions contacted, 32 were reached while the remaining eight were either non-existent or had moved. Out of a total of eleven participants, two were caring relatives while the remaining nine were staff members. | Most facilities were not designed for Turkish PWLD. Even in the few that did, professionals had limited interaction with them or their relatives.  Awareness needed that perception of dementia may be different due to culture (family) values and less information about available services influence participation. The use of community members who are trusted by the target group may be more effective for outreach. |
| Tezcan-Güntekin H.  (2018)  Germany | Expert interviews and problem-based interviews | Examine ethical conflicts that arise in the context of medical and nursing care for dementia patients with a migration background | N=12 | Hospital and home care providers. | **Technology-mediated Outreach and face-to-face Strategy:**  Participants were recruited through key stakeholders, such as doctors and nurses active in Turkish communities. | **Inclusive Practices and Cultural Considerations:** Potential Participants were informed in both languages (German and Turkish).  The interviews were conducted in German or in Turkish.  Bilingual posters, staff and information leaflets were used. | | Only caregiving relatives were involved, like spouses, adult children, or grandchildren. The age range was between 38 and 62 years. Two of the participants were male. | High heterogeneity of the included participants, low level of similarities within the Turkish participants.  Be aware of possible intercultural conflict within the care recipient and plwd.  Preconceptions (such as othering) may lead to conflicts between professionals, caregiving relatives and plwd. |
| Van der Heide et al. (2021)  Netherlands | Cluster Randomized Controlled Trial (RCT) | Understanding the health-related quality of life (HRQL) of family caregivers with Turkish or Moroccan immigrant backgrounds caring for people with dementia and assess the potential improvement in HRQL. | N= 180 | Through imams,  immigrant care organizations  regional center of Alzheimer Netherland in ethnic communities with relatively high number of individuals with Turkish or Moroccan backgrounds. | **Face-to-face Strategy:** Participants were contacted through key stakeholders (such as imams). If the person was interested they were contacted by the study coordinator to obtain eligibility. | **Inclusive Practices and Cultural Considerations:**  The HRQL questionnaires were available in Dutch and translated in Turkish and Moroccan-Arabic. | | In each region, there was one group with Turkish migrants and one group with Moroccan migrants. Only in one region, there were three groups of Turkish migrants and one group of Moroccan migrants included. The study analyzed 34 groups, with 16 groups consisting of Moroccan migrants and 18 groups consisting of Turkish migrants. | The Moroccan-Arabic version of the questionnaire was not chosen by any of the participants, in contrast, the Turkish version was predominantly used.  It is important to verify with the target population which language the prefer. |
| Van Wezel et al. (2016)  Netherlands | Qualitative Design with Semi-Structured Individual and Focus Group Interviews | Explore the perspectives of female Turkish, Moroccan, and Surinamese Creole immigrants about providing family care at home to a close relative with dementia and to identify similarities between the communities. | N= 16 | Community centers and home care provider. | **Face-to-face Strategy:**  Key figures from ethnic communities, dementia educators or care providers were asked to identify potential interviewees | **Inclusive Practices and Cultural Considerations:**  Most of the individual interviews were conducted in Dutch, except for six Turks. Individual interviews took place at the participant's home. The focus group interviews were held in a social or community center or care home, one was conducted in Turkish, one in Arabic and the rest in Dutch. | | The age range of the relatives was between 31 and 74 years.  Among the 16 participants, 6 of them were living close to the person affected by dementia. 14 of the participants were their daughter, one daughter-in-law and one spouse. | To care for a family member with dementia is embedded in the (cultural) value of family, involving professional care is linked to the fear of diminishing respect and appreciation. This may lead to being hesitant to participate in research.  The age of the female caregiver seems to have an impact on this perspective (younger women having a different/modern view on the involvement of professionals), which may lead to a different perception of getting involved research. |
| Van Wezel et al. (2022)  Netherlands | Cluster Randomized-Controlled Trial | Provide an overview of the Dementia Knowledge Scale's (DKS) development and validation among family caregivers with Turkish or Moroccan background. | N= 117 | In community centers in a large city in the south of the Netherlands. | **Face-to-face Strategy**: Participants were recruited through community centers (not part of the mains study)  Main study: recruited by key figures in the community, such as community workers, imams, ethnic-minority senior citizen adviser, ethnic minority care organizations, regional Alzheimer Association Netherland | **Inclusive Practices and Cultural Considerations:** The DKS Items were translated from English into Dutch and rephrased in a simpler language if needed. Translated items in Turkish and Moroccan Arabic were available.  Surveys were completed in a room at a mosque or a cultural community center | | **The Pilot Study:** 60 people with a Turkish or Moroccan background participated to test feasibility, comprehensibility and appropriateness of the DKS.  **Main Study**: Participants could choose whether they wanted to complete the questionnaire in Dutch or in Turkish/Moroccan Arabic. Three participants filled out the Moroccan version, one the Moroccan Arabic version and 16 completed the Dutch version of the DKS | Bilingual research assistants (Turkish or Moroccan) provided support with filling out questionnaires in case of problems (if needed the researcher read the information /DKS questions).  Participants with a Turkish background stated that they had greater reading and writing competence in Turkish than in Dutch. Participants with a Moroccan background, were more likely to choose the Dutch version than the Arabic version.  It is important to verify with the target population which language the prefer |
